# Compulsory treatment of physical illness under MHA 1983

**DOI:** 10.1136/medethics-2021-107438

**Published:** 2021-09-11

**Authors:** Robert Wheeler, Alexander Ruck Keene

**Affiliations:** 1 Paediatric Surgery, University Hospital Southampton NHS Foundation Trust, Southampton, UK; 2 39 Essex Chambers, London, UK; 3 Dickson Poon School of Law, Kings College London, UK

## Introduction

Taken together, Sections 145 & 63 of the Mental Health Act 1983 (MHA) provide for treatment without consent of physical illness ancillary to the mental disorder with which a patient presents. On a daily basis, clinicians make both the decision that the Act’s authority can be applied to their patient’s case, and whether or not it should be applied without the patient’s consent. But in the unusual circumstances where there is uncertainty as to the applicability of the MHA to the ancillary treatment of physical illness, the assistance of a court may be sought. In so doing, the law (and thence the courts) may justify compulsion if the treatment is within the scope of s63. But the law never prescribes it; the clinician is presented with authority that he or she *could* use but is left to decide whether it *should* be employed. This paper explores how the clinical question is set before the courts, and whether the distinction between symptom, manifestation and consequence is sufficiently understood by the courts. This is relevant to the context of self-neglect and its close cousin self-harm: the question whether the relevant ailment was caused by neglect or self- inflicted harm will determine whether compulsion under the MHA is applicable; and furthermore, whether or not compulsion is clinically acceptable.

## The case of CC, and the context of previous decisions

In the recent case of *CC*^1^ , a 34 year old man had suffered from type 1 diabetes since he was 15, and had complications of this due to his poor compliance with treatment, including retinal disease and soft tissue infection. At 28 he was informally admitted to hospital with a personality disorder; depression and features of psychosis. Six months later, he was detained under the MHA and has remained thus. In 2019, CC had presented with anaemia and was found to have renal failure, requiring subsequent haemodialysis. No medical evidence adduced to assert that his renal failure was either caused by the diabetes or exacerbated by his poor compliance appears in the court report, other than that of a psychiatrist who attributes the renal disease thus: ‘His diabetic control has been so poor for so long that he has significant physical sequelae of these (sic) including…renal failure…’. Only on this basis, the judge found that ‘The need for dialysis stems from CC’s self-neglect, including in regard to diet, which has led in whole or in part to his kidney failure. The reason his diabetes has resulted in kidney failure is to a large extent because of that self -neglect, which is itself a consequence of his mental disorder’.

His case had come to court to determine whether he could be compelled to have haemodialysis at times when he did not consent to it.

It was accepted by the court that CC had ‘at best’ fluctuating capacity to make decisions about his medical treatment, and at times had made it clear that he wanted to live. Nevertheless, at times of non-compliance he opposed treatment, probably lacking capacity in those phases. He ‘actively undermine[d] attempts to manage both of these conditions and regularly refuses treatment for them’. One issue for the court to decide was whether ss.63/145(4) MHA could be used to authorise compulsory haemodialysis.

CC’s consultant psychiatrist considered that CC’s non-compliance with dialysis was a *manifestation or symptom* of his mental disorder; that his need for dialysis was a *consequence* of his mental disorders; and that he should be thus treated.

The judge, Mrs Justice Lieven, cited the judgment in *Nottinghamshire Healthcare NHST v RC* ^2^ noting that ‘…the extent to which a condition is within the ambit of s63 read with s145 can be difficult to ascertain’. In CC’s case, however, she considered that: ‘…this is a clear case…of dialysis treating a manifestation of …personality disorder’. She acknowledged that ‘…as with any mental disorder…and its link to physical ill-health, causation and the link between the mental and physical condition is intensely complex and not really amenable to a primary cause analysis…..however it seems to me clear that the physical condition CC is now in (renal failure)…is properly described as a manifestation of his mental disorder’. The effect of the judgment from CC’s point of view was that his consent was not required for dialysis during the duration of his detention under the MHA. Given the judge’s reasoning, and since neglect of his diabetes was held to be a symptom of his mental disorder, it follows that a compulsory insulin injection regime, regular blood glucose monitoring, dietary control of carbohydrates, toenail management (to prevent sepsis) and ophthalmic interventions to preserve CC’s sight would also be possible under the compulsion of s63, capacitous or otherwise. However, it seems unlikely that clinicians would choose to employ compulsion in the areas of self-neglect that had no causative bearing on CC’s renal failure, given that the case was pleaded only on the basis that renal replacement therapy was required. It matters that CC had fluctuating capacity. When capacitous, presenting a transient window into his wishes and feelings, he chose to live. If he had had unremitting incapacity which had not been explicable by the illness that made him eligible to be detained in the first place, then the MCA 2005 would have instead been available as an alternative authority for compulsory treatment.

As a separate point, of importance to patients whose fate is to be determined by the court, this case illustrates how judicial assumptions of causation may emerge founded on incomplete medical evidence. Whilst it remains likely that CC’s renal disease flowed exclusively from diabetes, it is also possible that in his case the two were unrelated. Whilst diabetics can acquire renal failure as a consequence of their diabetes, they can also suffer this condition due to causes entirely unrelated to diabetes. It would have been comforting to have had assurance that the judge was provided with that information, if only to dismiss it.

The judge in CC indicated that s63/145 MHA 1983 was not ‘amenable’ to judicial analysis. Arguably, the courts are not really engaged in restricting the power to treat. With one partial exception (the *GJ* case discussed below), we cannot identify a case where a court has held treatment to be outside the relevant statutory provision in circumstances where the patient was found to have a mental disorder.

By contrast, in many cases the courts have taken exceptionally broad view of what sort of compulsion is lawful. A Caesarean section ^3^ was found to be within s 63 as a treatment for schizophrenia, since the patient would have been caused undue distress if her baby had come to harm. In *B v Croydon*^4^, a court pondered whether a woman with a personality disorder might be allowed to refuse food, but ultimately found that she could be treated under the MHA without her consent. Ian Brady^5^ , sentenced to three concurrent terms of life imprisonment, was on hunger strike. He sought judicial review of whether the consequent decision to force feed him in the secure hospital where he was accommodated was irrational; and whether it could be justified under s 63, contending that it was not medical treatment for a the mental disorder from which he was suffering. Framing the judgement in terms of whether the medical officer’s decision to treat using the justification of s63 was reasonable, the court was satisfied ‘…to a high degree of probability…’ that it was. Maurice Kay J held that Brady’s hunger strike was a manifestation or symptom of his personality disorder, and the feeding was necessitated by his state of starvation. The judgement noted Brady’s profound fear of returning to prison and some of the steps taken to protect him; Brady’s crimes were notorious and widely reviled. In the final paragraph of his judgement Maurice Kay J concluded that he was entirely satisfied that ‘…the decision to commence and continue force feeding was justified by reference to s63 and that it is and was and is in all respects lawful, rational and fair.’ Whilst lawfulness and irrationality were central to the application for judicial review, ‘fairness’ played no part in the pleadings, or in the statutory determination. The operation of fairness perhaps should be considered in the context of the crime for which Brady had committed, and his refusal to offer the bereaved families solace by revealing where their children’s bodies lay. There was no evidence placed by the medical staff before the court that indicated a disinclination to use s63 as an authority to administer force feeding.

Although – as in *CC* – the courts continue to accept that s63 has a potentially very broad definition, it is also important to note that clinicians are now less regularly asking the courts to include treatments within its scope. This can, perhaps, be tied to the coming into force of the Mental Capacity Act 2005, which provides a very clear alternative framework for thinking about treatment. By way of example, in *Dr A’s* case,^6^ the hospital was faced with the need to provide artificial nutrition and hydration. However, Dr A’s psychiatrist did not agree that food was treatment for her patient’s mental illness. Dr A was a 50 year old Iranian doctor seeking asylum in the UK. His applications had been refused, and his Iranian passport had been withheld by the UK Border Agency to be returned on condition that he returned to Iran. He had embarked on a hunger strike in an attempt to recover his passport, but became ill and developed paranoid and conspiratorial ideation. Dr A’s psychiatrist saw his stance as founded on a credible political stance rather than an egregious act of criminality. However, she agreed that treatment was legitimate, and the Trust brought the case to court. Baker J held that Dr A lacked capacity to make decisions about nutrition and hydration because his delusional illness affected his ability to use or weigh the information relevant to the choice to accept nourishment. The judge found that Dr A’s physical disorder was not obviously a ‘…manifestation or symptom of his mental disorder even though it was a consequence of it’. Whilst the necessary force feeding could therefore not be administered under s 145, it could be authorised under the inherent jurisdiction^i^, and this was the action taken by the court.

During her final submissions on behalf of the Trust, counsel set out the clinical concerns of reliance on s63 to treat physical conditions, not least that this would increase the number of patients who will perforce be treated compulsorily under the MHA. Counsel noted that such a development would be ‘therapeutically undesirable’, since there was a strong clinical preference to treat patients informally under the Act because of the hope that a patient acting voluntarily is more likely to engage with treatment. From the clinicians’ perspective, an application to the Court of Protection (under the MCA) or the High Court (in the case of the inherent jurisdiction) seeking an order in the patient’s best interests was a far more nimble way of providing a remedy for a patient opposing force feeding than the ‘blunt’ instruments of challenging a s3 or seeking judicial review.

## Symptoms, manifestations and consequences

The simple – but stark – question that these judgments pose: Is this heterogeneity of statutory interpretation what Parliament intended for the use of the MHA? And, closely related to this, what are the implications of these judgments for the legal response to self-neglect?

For clinicians, it is natural to view this as a clinical question, turning on fairly close readings of the terms ‘symptom’ and ‘manifestation’. Symptoms and their cousins, manifestations (a display of existence), indicate a characteristic sign of a particular disease. They accompany that disease and provide evidence of its presence. ‘Consequences’ follow closely the natural unfolding history of a disease but are a different entity, since they give no specific indication of underlying diagnosis. As the case of CC demonstrates, renal failure can be a consequence of a mental disorder, but cannot by itself provide evidence for the presence of mental disorder. But it is evident that Parliamentarians view the words differently: Baroness Royall, speaking for the Government on Commons Amendments to the Mental Health Bill in 2007, in the context of proposed amendments to the definition of s145, 'broadly speaking' considered that ‘…‘symptoms’ covers the consequences of which patients themselves complain while manifestations more obviously cover the evidence of the disorder as seen by other people’. Chris Bryant MP took a narrower approach:

‘I have insisted on the phrase “symptoms and manifestations” in amendment No. 3 because I believe that the manifestation of an element of a mental disorder must have a direct relationship with that disorder. The relationship cannot be indirect; the manifestation must, by definition, be an element of the condition. The reason for the word “manifestation” is that it has a direct route into the mental disorder. Let me give an example. Somebody who is schizophrenic might suffer and be extremely angry because he has just split up with his partner. He might be furious with her. However, we should not be allowed to detain somebody solely to treat that anger unless the perhaps violent anger is a direct manifestation of the mental disorder from which he suffers. That is important.’^7^

It is therefore not obvious that the legislature viewed ‘symptoms’ and ‘manifestations’ as distinctly different. The central issue is the causal nexus between the mental illness and the clinical picture. Perhaps clinicians interpret these words too closely; seeking to make medical what is properly a legal question in circumstances where the intent of Parliament appears to have been to restrict the application of the section to conditions directly and closely related to the mental disorder for which the individual was detained.

If right, this raises the question whether how self-neglect that causes sufficient harm to merit compulsory treatment should be characterised.

## Self-neglect and the refusal of medical treatment

The courts have been quick in the past to see irresponsible decisions taken by the patient as a manifestation of mental disorder. The judgments in *Brady* and *Dr A* illustrate the complexities of evidence placed before the courts. In such serious clinical situations, the courts tend to read any provision expansively in favour of the doctor’s authority to treat under compulsion.

From a clinical perspective, self-neglect can undoubtedly be described as both a symptom and a frequent manifestation of mental disorder. However, it would be an insidiously authoritarian step to propose that anything other than self-neglect causing immediate serious physical harm should legitimate the use of detention and treatment under s63 MHA 1983. Conversely, simply **not** treating could be equally problematic.^ii^

It seems that the current law is not a meaningful barrier to compulsory treatment for a very wide array of physical conditions. Nor, by the same token, is it a very helpful guide as to what clinicians should do. We can either see that as a problem, or as the opportunity for clinical practice to develop its own ethical precepts. The discussion of self-neglect could usefully be a starting point for that development, and, to illustrate how, we set out a schema for thinking through the response to renal failure in the context of self-neglect:

- If the person has capacity to make decisions about medical treatment, but ‘cannot help’ himself by adhering to necessary treatment then it may be legitimate to detain them under the MHA 1983, and it may also be legitimate for s.63 to be used to compel dialysis. This would be on the basis that interim relief would enable the person, once his mental disorder had abated with medical treatment, once more to give priority to renal health. The basis for compulsion for both medical treatment for mental disorder and renal treatment would both coincide and evaporate simultaneously. The permissive nature of the declaration that would be made by a court in any application (as in CC’s case) would leave the door open to clinical discretion to decide whether the team should use compulsion.
- If the person lacks capacity to make decisions about medical treatment, then either the MHA or the MCA will be in play as a potential treatment framework. The critical question will be whether their inability to make the decision is because of a condition amenable to medical treatment for mental disorder. In *GJ v Foundation Trust* [2009] EWHC 2972, the patient forgot to take his insulin because of his dementia, rather than because of a condition amenable to medical treatment under the MHA 1983. The court found therefore that, despite his objections, the appropriate regime for securing his treatment was the MCA, under the so-called Deprivation of Liberty Regime.
- In either case, however, if the medical treatment is not going to improve the person’s situation, it should not be given either under the MHA or the MCA. It is difficult to see that it is in the patient’s best interests under the MCA^8^ or that it would be clinically justified under the MHA.

To take another example (from the *RC* case) we suggest that the anaemia caused by the slashing open of a brachial artery by a person with personality disorder seems to be precisely the sort of problem that can properly be included in the MHA ambit. Beyond a requirement of therapeutic appropriateness, we do not ask whether there will be permanent or long-term improvement (if he continues to cut herself, there may well not be). However, there must also be some longer term treatment plan for the patient; otherwise there would be no proper basis for the patient to be detained.

## Conclusion

The proposed reforms to the MHA^9^ (which in turn respond to the Wessely Review)^10^ would give patients more rights to challenge detentions, and expand the powers of tribunals so that they play a greater role in embedding both therapeutic benefit and the principle of least restriction. While the proposals stop short of giving the tribunal powers to order treatments, it does seem that if the amendments proceed, there will be considerable additional scrutiny of clinical decision-making as regards compulsory treatment. This means that it is all the more important that clinicians both develop and are prepared to explain clear bases for their decisions in this context.

With specific reference to self-neglect, we suggest that it would be helpful if medical experts are ready clearly to identify symptoms, manifestations and consequences of disease; and to distinguish the repercussions of acts of self-neglect (which cause physical illness that require to be treated by compulsion), from those aspects of self-neglect that have no bearing on the relevant physical illness. Neglectful or otherwise, a capacitous patient, in the absence of mental illness, is fully entitled to refuse treatment. On the other hand, self-neglect in patients with mental illness is commonplace. The consequences of self-neglect are illustrated in CC, and are equally applicable to the countless patients in whom a mental disorder coexists with chronic serious physical disease, which can threaten life if neglected. It is therefore important, in patients with physical illness who are liable to compulsory treatment under the MHA to identify those in whom *self-neglect of physical illness is causing harm*. As the judgment in CC has illustrated, whether construed as self- neglect, symptom, manifestation or consequence, concomitant serious disease causing harm in the context of mental disorder makes ss.145/63 available for the treatment of these diseases.

Equally, clinicians seeking declarations of powers of compulsion authorised by the MHA must remain alive to the notion that whilst the courts are equipped to declare that compulsion *could* be used, it remains ultimately a clinical matter (informed by appropriate ethical consideration) as to whether it *should* be employed.

## Footnotes

i For technical legal reasons, the MCA 2005 route could not be used, but Baker J applied the same tests of capacity and best interests that he would have done had been applying that Act.

ii For a vivid example, see Legal literacy a factor in death of man who refused medical help (communitycare.co.uk).

1 A Healthcare & B NHST v CC [2020] EWHC 574 (Fam)

2 Nottinghamshire Healthcare NHST v RC [2014] EWCOP 1317

3 C v Rochdale [1997] 1 F.C.R. 274

4 B v Croydon HA [1995] 1 All ER 683 (CA)

5 R v Collins & Ashworth Hospital Authority, ex p Brady [2000] LLR Med 355

6 An NHS Trust v Dr A (By his litigation friend the Official Solicitor) [2013] EWHC 2442 COP

7 Hansard, HC, 461, 1280, 18^th^ June 2007

8 University Hospital Birmingham NHS Foundation Trust v AI & K [2021] EWCOP 37

9 https://assets.publishing.service.gov.uk/government/uploads/system/uploads/attachment_data/file/778897/Modernising_the_Mental_Health_Act_-_increasing_choice__reducing_compulsion.pdf accessed 8^th^ June 2021

10 https://www.gov.uk/government/consultations/reforming-the-mental-health-act/reforming-the-mental-health-act accessed 8^th^ June 2021

